# Diselenide Core Cross-Linked Micelles of Poly(Ethylene Oxide)-*b*-Poly(Glycidyl Methacrylate) Prepared through Alkyne-Azide Click Chemistry as a Near-Infrared Controlled Drug Delivery System

**DOI:** 10.3390/ma13122846

**Published:** 2020-06-25

**Authors:** Sonita A.P. Siboro, Sabrina Aufar Salma, Hyeung-Rak Kim, Yeon Tae Jeong, Yeong-Soon Gal, Kwon Taek Lim

**Affiliations:** 1 Department of Display Engineering, Pukyong National University, Busan 48513, Korea; sonita.afrita@gmail.com (S.A.P.S.); sabrinaaufarsalma@gmail.com (S.A.S.); ytjeong@pknu.ac.kr (Y.T.J.); 2 Department of Food Science and Nutrition, Pukyong National University, Busan 48513, Korea; hrkim@pknu.ac.kr; 3 Department of Fire Safety, Kyungil University, Gyeongsan 34828, Korea; ysgal@kiu.ac.kr

**Keywords:** NIR-responsive, diselenide bond, CCL micelles, indocyanine green

## Abstract

In this article, a drug delivery system with a near-infrared (NIR) light-responsive feature was successfully prepared using a block copolymer poly(ethylene oxide)-*b*-poly(glycidyl methacrylate)-azide (PEO-*b*-PGMA-N_3_) and a cross-linker containing a Se-Se bond through “click” chemistry. Doxorubicin (DOX) was loaded into the core-cross-linked (CCL) micelles of the block copolymer along with indocyanine green (ICG) as a generator of reactive oxygen species (ROS). During NIR light exposure, ROS were generated by ICG and attacked the Se-Se bond of the cross-linker, leading to de-crosslinking of the CCL micelles. After NIR irradiation, the CCL micelles were continuously disrupted, which can be a good indication for effective drug release. Photothermal analysis showed that the temperature elevation during NIR exposure was negligible, thus safe for normal cells. In vitro drug release tests demonstrated that the drug release from diselenide CCL micelles could be controlled by NIR irradiation and affected by the acidity of the environment.

## 1. Introduction

Nowadays, the core-shell structures of polymeric micelles have attracted much attention in the field of drug delivery systems due to their high loading capacity [1,2] and hydrophobic drug solubility [3,4]. These typical properties owe to the presence of an internal core and their amphiphilic nature. A block copolymer consisting of polar and nonpolar chains, which are fabricated from hydrophobic and hydrophilic polymers, usually builds up kinds of micelles [5,6,7,8,9]. However, the sole appearance of Van der Waals interactions at these self-assembled polymeric micelles leads to low stability, causing premature drug release [10,11]. This circumstance not only generates inefficient drug delivery but also can jeopardize normal tissues. Thus, improving the stability of the core-shell structure of polymeric micelles is essential in order to obtain an ideal drug delivery system.

To enhance the stability of polymeric micelles, the use of a cross-linker is considered as the most effective method [12,13]. In typical core-cross-linked (CCL) micelles, not only does the stability become significantly increased, but also drug release can be controlled by applying certain stimuli [14]. Depending on the chemical properties of cross-linkers, drug release in CCL micelles can be governed by several intracellular and/or extracellular factors. Recently, some publications reported the success of controlled drug release by utilizing physicochemical properties such as pH-responsive [15,16,17], redox-responsive [18,19], temperature-responsive [20,21,22], and even light-responsive [23,24]. In fact, stable polymeric micelles with responsive release ability are now critically demanded as drug delivery materials. 

Among several stimuli that can trigger a release of drugs from their carrier, light—especially near-infrared (NIR) light which possesses relatively low energy—has advantages as non-contact stimuli, and thus can be safe for human cells. Recently, our group has reported a successful synthesis of NIR-responsive drug delivery employing a diselenide-containing cross-linker [25]. The Se–Se bond in the diselenide cross-linker shows relatively low bond dissociation energy (Se–Se = 172 kJ mol^−1^) compared to its analog of a disulfide bond (S–S 240 kJ mol^−1^), thus the diselenide bond is more susceptible to mild stimuli. Furthermore, selenium also exhibits good biocompatibility and low cytotoxicity, thus it is safe for the human body [26,27]. Considering these facts, it is crucial to carry out further development of NIR-responsive drug delivery based on diselenide CCL micelles. 

**Scheme 1:**
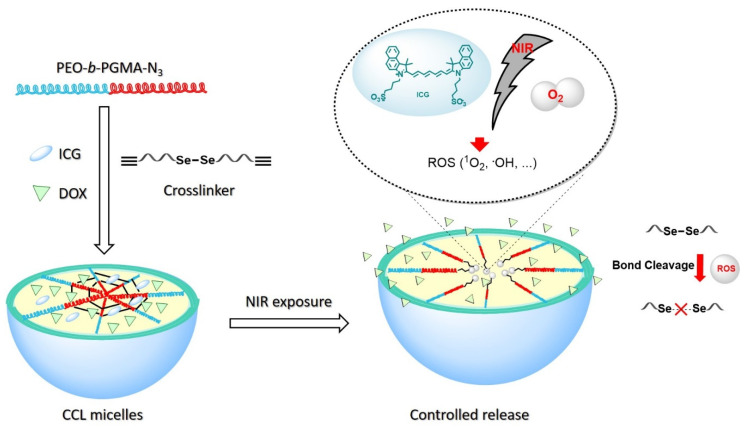
Near-infrared (NIR)-controlled drug release.

In this contribution, we successfully developed another variant of diselenide CCL micelles by employing alkyne-azide click chemistry. A block copolymer poly(ethylene oxide)-b-poly(glycidyl methacrylate (PEO-*b*-PGMA) was synthesized via single electron transfer radical living polymerization and subsequently functionalized by azide groups. The click reaction between diselenide cross-linkers and azide groups in the polymer backbone was easily carried out, resulting in core-cross-linked (CCL) micelles with NIR-responsive features. In the presence of ICG, NIR exposure triggered the production of reactive oxygen species (ROS). ROS attacked the diselenide bond, causing the micelles to disassemble and release the drug molecules. Therefore, over this strategy, NIR-controlled drug release can be achieved as illustrated in Scheme 1.

## 2. Results and Discussion

### 2.1. Preparation of Diselenide Cross-Linkers

**Scheme 2:**
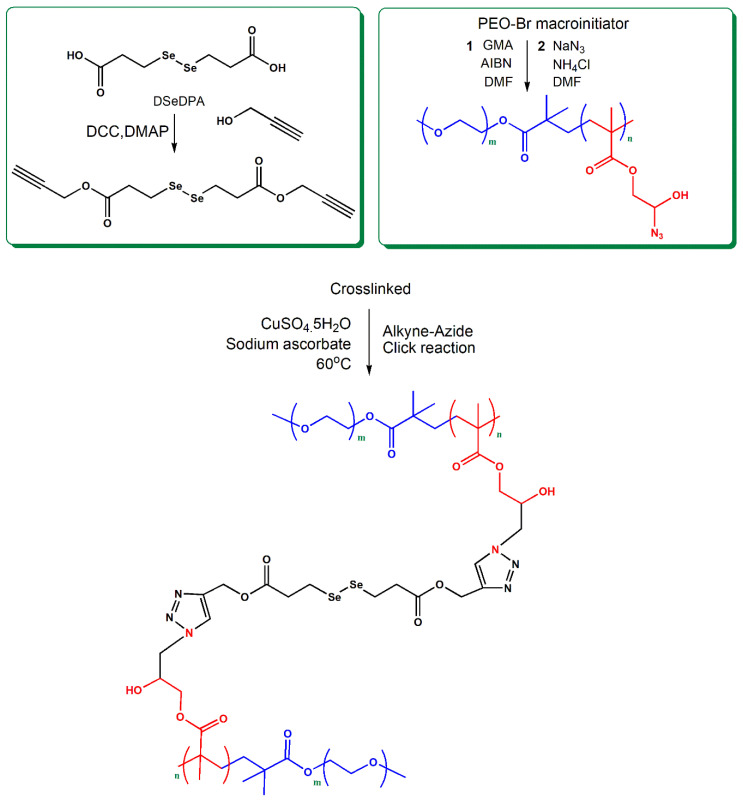
Preparation of diselenide CCL micelles.

In our previous report, the diselenide-containing cross-linker was successfully synthesized via preparation of 3,3’-diselanediyldipropanoic acid (DSeDPA) and subsequently reacted with hydroxyethyl maleimide, resulting in the maleimide bifunctional diselenide cross-linker [25]. In this current experiment, a similar procedure was conducted to synthesize DSeDPA, and it was subsequently reacted with alkyne groups to produce alkyne bifunctional diselenide cross-linkers. The alkyne bifunctional diselenide cross-linker was then introduced to the amphiphilic block copolymer of poly(ethylene oxide)-b-poly(glycidyl methacrylate)-azide (PEO-*b*-PGMA-N_3_) using CuACC strategy. Indocyanine green (ICG) and doxorubicin (DOX) were then loaded into the CCL micelles. ICG was expected to produce ROS under NIR exposure to break the diselenide bonds, thus the drug can be released under external control (Scheme 2).

**Figure 1:**
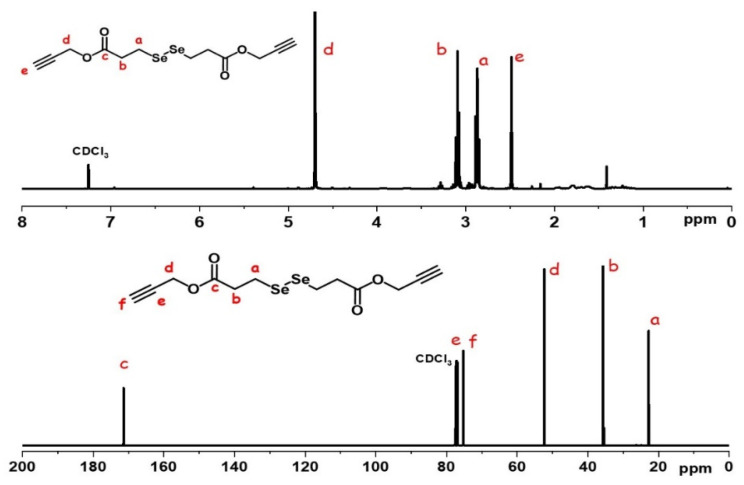
^1^H NMR (top) and ^13^C NMR (bottom) of diselenide cross-linker.

As shown in Figure 1, the proton peak of the alkyne group is shown at 2.49 ppm as a singlet and the protons of methylene in the ester bond appear at 4.70 ppm. The protons of methylene groups adjacent to the diselenide bond appear at 2.85 ppm in triplet peaks. The ^13^C-NMR spectra demonstrate the carbon peaks of the ester carbonyl peak at 171.95 ppm, the alkyne group at 76.83 ppm, and 78.46 ppm, and the ester methylene carbon peaks at 36.65 ppm. The methylene group carbon adjacent to the Se-Se bond appears at 23.25 ppm. These results show that the diselenide cross-linker with the bifunctional alkyne groups is successfully synthesized. 

### 2.2. Preparation and Characterization of CCL Micelles

**Figure 2:**
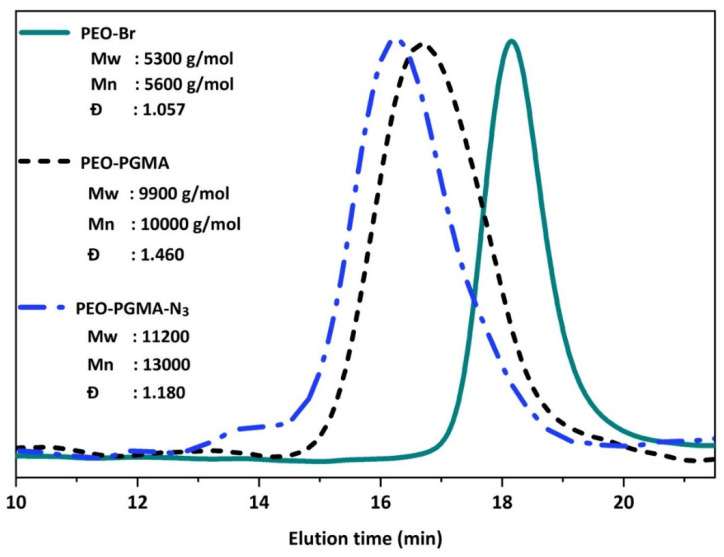
GPC chromatogram of PEO-Br, PEO-*b*-PGMA and PEO-*b*-PGMA-N_3._

The block copolymer PEO-*b*-PGMA was synthesized via the single electron transfer living radical polymerization of GMA with a PEO-Br macroinitiator. As shown by Gel Permeation Chromatography (GPC) analysis (Figure 2), the molecular weight (M_w_) of the product could be well controlled with a narrow dispersity (Đ), indicating the polymerization was quite suitable for this process. It was confirmed that the M_w_ of the initial PEO-Br was 5300 g/mol with Đ of 1.057, and the M_w_ of the PEO-*b*-PGMA block copolymer after polymerization was 9900 g/mol with Đ of 1.46. Subsequently, the azide group was attached to the block copolymer backbone through the ring-opening of the epoxide moieties. After the incorporation of the azide groups, the molecular weight was slightly increased as shown in the GPC elution time.

**Figure 3:**
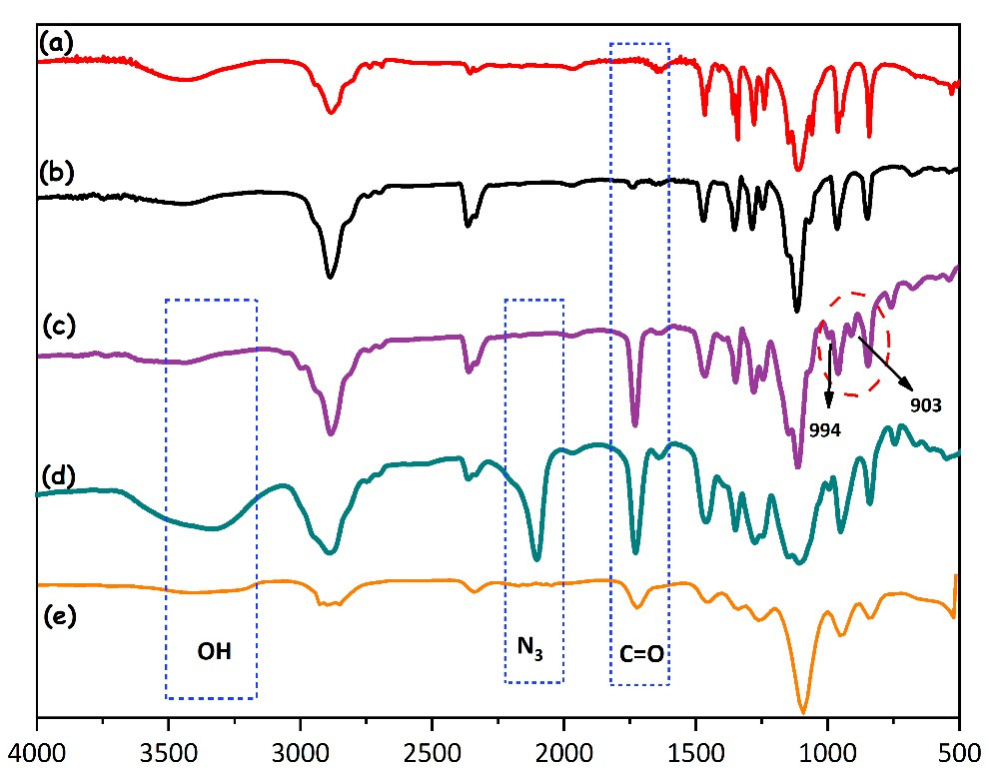
FTIR spectra of (**a**) PEO, (**b**) PEO-Br macroinitiator, (**c**) PEO-*b*-PGMA block copolymer (**d**) PEO-*b*-PGMA-N_3_, and (**e**) CCL micelles of PEO-*b*-PGMA.

The preparation of the polymers and formation of the CCL micelles were analyzed using FTIR spectroscopy. The presence of the macroinitiator moiety in the PEO polymer was indicated by the appearance of the carbonyl vibration band at 1735 cm^−1^ (Figure 3b). In Figure 3c, the vibration band at 903 cm^−1^ and 994 cm^−1^ corresponded to the vibration of the epoxy ring in PEO-*b*-PGMA block copolymers. The methyl and methylene of the PGMA should exhibit a vibration band at 2800–3000 cm^−1^. Nonetheless, these typical vibrations overlapped to the methylene band out of the PEO-Br macroinitiator, therefore they were not observed separately in the FTIR spectra (Figure 3b,c). Functionalization of the block copolymer PEO-*b*-PGMA by azide groups was identified by the appearance of a new peak at 2090 cm^−1^, which can be attributed to the azide functional vibration in PGMA-N_3_ moieties (Figure 3d). Moreover, the opening of epoxy rings has generated a broader and more intense band of O-H group at 3450 cm^−1^, while the typical peaks of the ring at 903 and 994 cm^−1^ seem to disappear. After alkyne-azide click reaction, the typical spectra of the azide vibration band was significantly reduced, showing that the reaction between alkynes from the diselenide cross-linker and azides from PGMA-N_3_ was successfully achieved (Figure 3e).

**Figure 4:**
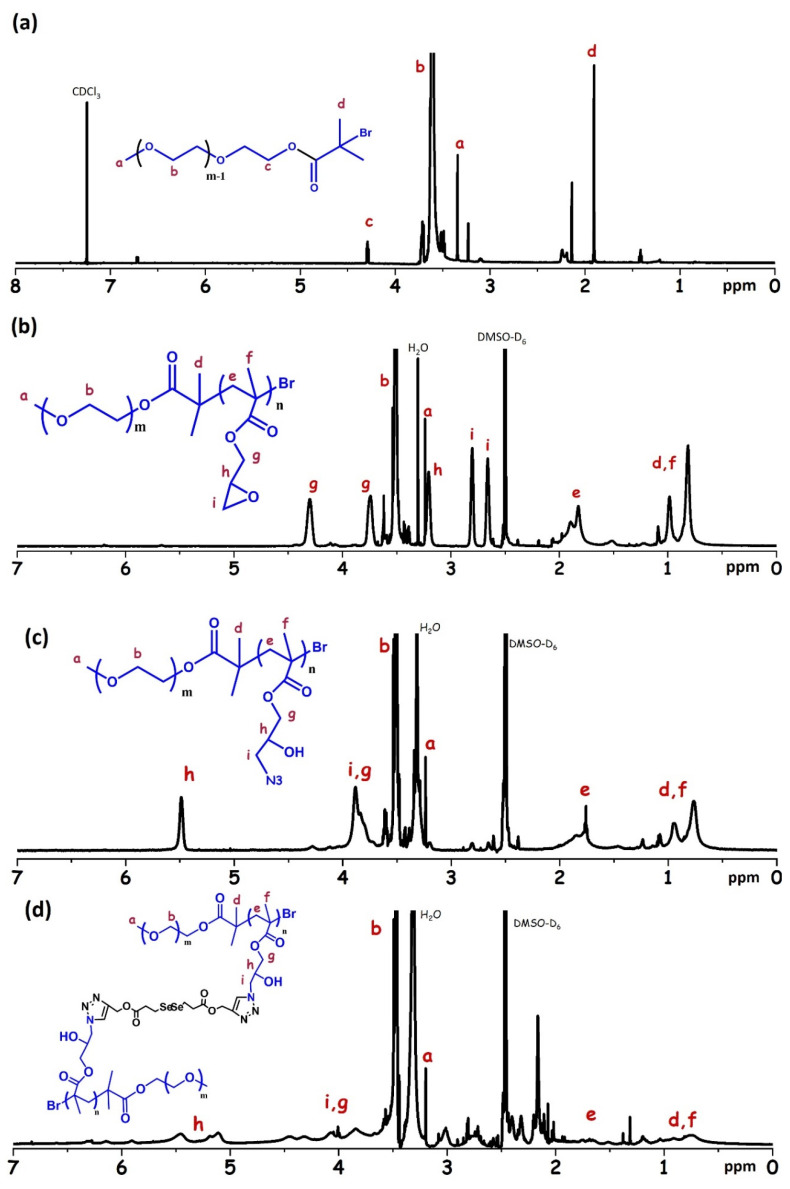
^1^H NMR of (**a**) PEO-Br macroinitiator, (**b**) PEO-*b*-PGMA block copolymer, (**c**) PEO-*b*-PGMA-N_3_, and (**d**) diselenide CCL micelle of PEO-*b*-PGMA.

The polymer structure and the formation of CCL micelles were further confirmed by ^1^H NMR analysis. In Figure 4a, new peaks at 1.93 ppm and 4.32 ppm corresponded to methylene groups adjacent to the ester linkage and methyl groups of α-bromoisobutyryl bromide. These new signals confirmed a substitution of hydroxyl groups in the terminal PEO chain, demonstrating the presence of the PEO-Br macroinitiator. The protons of the methylene in the main chain of PEO appeared at 3.51 ppm, whereas the methylene protons (*s, t*) of the epoxy ring were detected at 3.21, 2.81, and 2.67 ppm. The proton of the methylene and methyl in PGMA moieties were shown at 4.31, 3.73, 1.90, 1.82, 1.20, 0.98, and 0.81 ppm, respectively (Figure 4b). These results confirmed that the PEO-*b*-PGMA block copolymer has been successfully prepared. After azidation process, the typical signals of the PEO chain were preserved, while the peaks of PGMA moieties were slightly altered (Figure 4c). The new peaks at 3.88 and 5.51 ppm can be attributed to the proton of CH_2_-N_3_, CH-O (s), and CH_2_-O (r), which showed that the ring-opening process has been achieved. These results confirmed that the azidation of the PGMA backbone was successfully carried out. After the click reaction of two alkyne groups from diselenide cross-linker and azide groups in the block copolymer PEO-*b*-PGMA-N_3_, the proton signals of PGMA-N_3_ were reduced significantly (Figure 4d). The click reaction promotes the formation of CCL micelles, which is comprised of PEO as a shell and PGMA as a core. As the PGMA parts were inside the micelles, their response to the magnetic field should be decreased (more shielded), resulting in depletion on the ^1^H NMR spectra. Therefore, the depletion of PGMA peaks strongly indicated that CCL micelles of the amphiphilic block copolymer were successfully formed by the diselenide cross-linker. 

**Figure 5:**
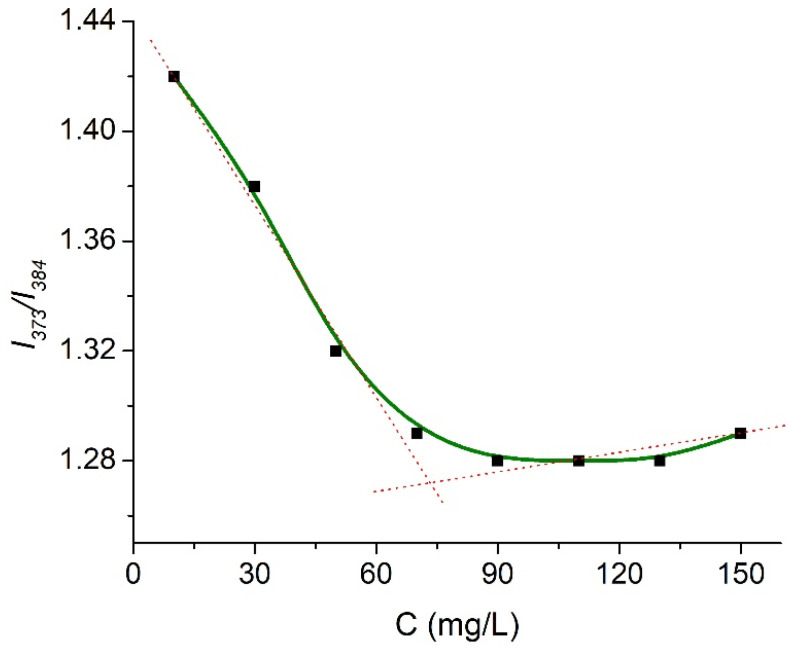
A graph of fluorescence intensity ratio versus polymer solution concentrations to determine the critical micelles concentration (CMC).

**Figure 6:**
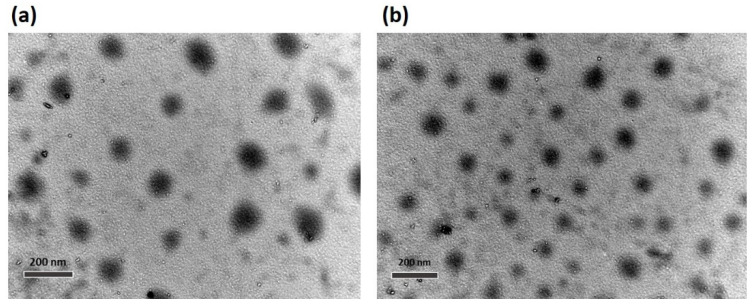
TEM images of (**a**) non-CCL micelles and (**b**) CCL micelles.

**Figure 7:**
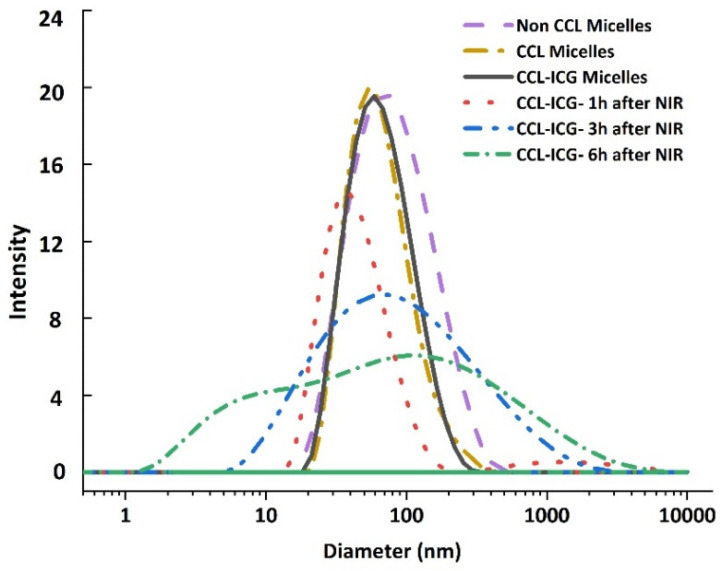
The hydrodynamic diameter of CCL micelles and non-CCL micelles measured using DLS.

The critical micelle concentration (CMC) was determined using a fluorescence spectrophotometer with pyrene as a probe. Figure 5 shows the graph plot between the fluorescence intensity ratio of pyrene peaks at 373 nm to 384 nm versus the concentration of the polymer solution. The CMC value was observed at the polymer concentration of 69.2 mg/L. The micelle concentration for further investigation was prepared at above the CMC value. The morphology and the size of the CCL micelles compared to non-CCL micelles were observed using TEM analysis (Figure 6). As can be seen, both CCL and non-CCL micelles have spherical shapes, but they exhibited different sizes. The average diameter of the CCL micelles (93 nm) was smaller than that of non-CCL micelles (109 nm). On the CCL micelles, the crosslinking reaction shrank the particle size, owing to the more compact core structure. For the further investigation of the hydrodynamic diameters of non-CCL micelles and CCL micelles, DLS analysis was performed (Figure 7). As shown, the non-CCL micelles and CCL micelles exhibited particle sizes of 114 nm and 96 nm, respectively, which was in good agreement with those from TEM analysis. After the loading of ICG, the size of the CCL micelles was only slightly changed, suggesting that the ICG was physically loaded into the micelles

### 2.3. NIR Light Irradiation to the CCL Micelles

The effect of NIR light irradiation on the behavior of CCL micelles was investigated using DLS analysis (Figure 7). The CCL micelles containing ICG were exposed to NIR light and analyzed using DLS after 1, 3, and 6 h, respectively. It was clearly shown that by prolonging the observation time, the hydrodynamic diameter significantly decreased and the DLS peaks became broader. This result demonstrated that the CCL micelles were disrupted by NIR exposure and the disruption continued by prolonging the time. Our latest report showed that the prolonged damage of micelles was attributed to not only Se–Se bond-breaking but also a degradation of the polymer chain itself [28]. In this case, the excessive ROS attacked PEO backbones and caused slow degradation over time. In addition, our previous study also showed that NIR exposure to micelles in the absence of ICG did not exhibit any effect [28]. Thus, only the behavior of ICG loaded micelles was presented in the current study.

**Figure 8:**
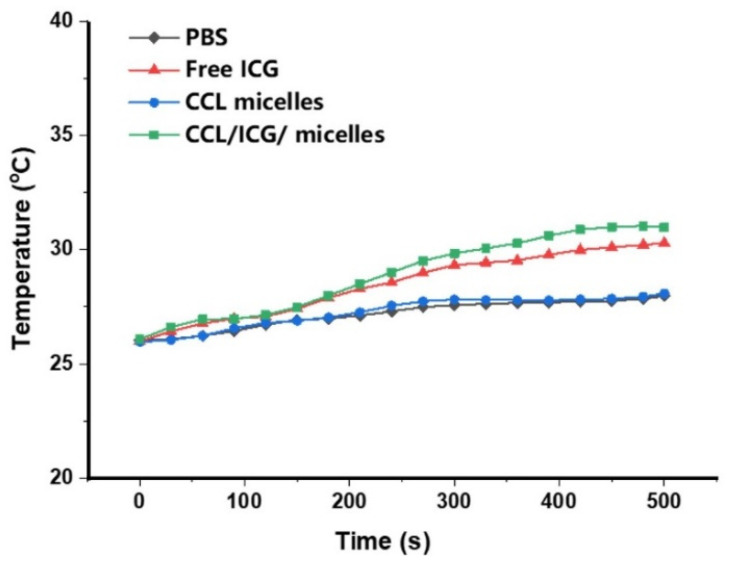
Profiles of temperature alteration of PBS, free ICG, CCL micelles, and CCL/ICG micelles as exposed by NIR light.

To obtain more insight into the phenomenon inside CCL micelles during NIR exposure, a photothermal analysis was carried out. The photothermal analysis was performed to PBS, free-ICG, CCL micelles, and CCL/ICG micelles (Figure 8). During NIR exposure, the temperature of free ICG and CCL/ICG micelles slightly increased (from room temperature to 30.2 and 31.1 °C, respectively). The temperature increase of CCL/ICG micelles was a little higher than that of free ICG, which indicates the presence of another reaction after the ROS has been produced. Nevertheless, this temperature increase is too small to damage cells, thus it can be concluded that the main role of ICG is only for generating ROS. The ROS then started breaking the diselenide bond that led to de-cross-linked CCL micelles as well as causing damage in the PEO backbone in a prolonged time. The temperature elevation in PBS and CCL micelles was negligible, suggesting there is no effect of the NIR exposure to the drug delivery materials except ICG.

### 2.4. Drug Release Behavior

The drug release behavior from DOX/ICG loaded CCL micelles was studied in several physiological conditions. The pH condition was controlled at 5 and 7.4, which simulated the pH of cancer cells and normal cells, respectively. Phosphate-buffered saline (PBS) was used as a solution to control the pH. Under both of these pH conditions, drug release behavior with or without NIR exposure was investigated.

**Figure 9:**
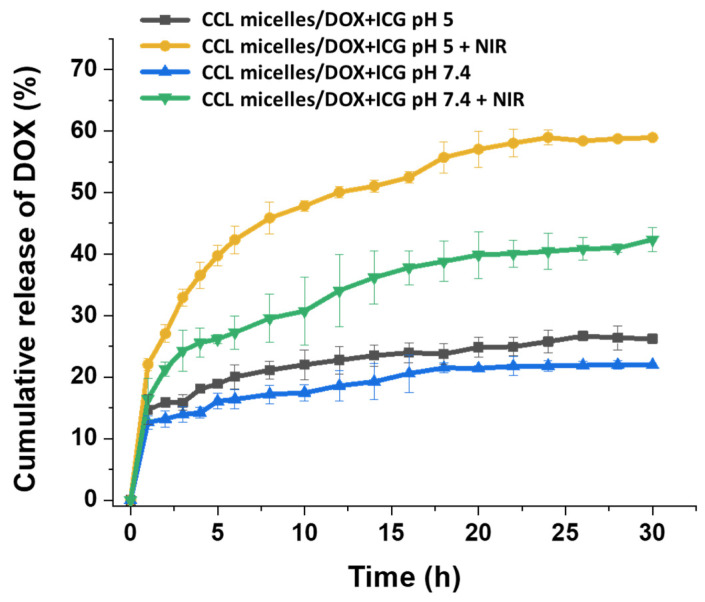
DOX release profile (cumulative) from CCL/ICG/DOX micelles with and without NIR irradiation in pH of 7.4 and 5.

As can be seen in Figure 9, CCL micelles at pH of 5 and 7.4 without NIR exposure exhibit lower drug release behavior during 30 h of observation. At pH of 5, the maximum drug release was 25%, whereas at pH of 7.4 the maximum drug release was only 20%. Since DOX has better solubility in acidic solution, this result can be clearly understood. Under the NIR exposure, the drug release behavior was increased dramatically. After 30 h, the maximum drug release at pH of 5 was 60%, which is more than two times than the drug release without NIR exposure. At pH of 7.4, the maximum drug release (40%) was slightly lower than that of pH 5. These results demonstrated that the drug release of diselenide CCL micelles could be controlled by using NIR exposure. The NIR exposure triggers ICG to produce ROS and the ROS then attacks the diselenide bond in the cross-linker. The cleavage of the diselenide bond leads to the de-crosslinking of CCL micelles and loosen the core structure. Moreover, ROS also attacks poly(ethylene glycol) chains, resulting in the polymer degradation [28]. This concerted mechanism accelerates the release of the DOX entrapped in CCL micelles. 

## 3. Conclusions

In this work, the diselenide CCL micelles of PEO-*b*-PGMA block copolymers were successfully prepared via single electron transfer living radical polymerization. The amphiphilic block copolymer was prepared by single electron transfer radical living polymerization. By incorporating ICG and DOX into the CCL micelles, the drug release can be triggered by NIR irradiation. During NIR irradiation, the ICG produced ROS, and the ROS caused the cleavage of diselenide bonds, leading to de-crosslinked CCL micelles. The CCL micelles were steadily disrupted after being exposed to NIR light. This could be a good sign of an effective and controllable drug release. Moreover, the system was considered safe to normal cells with regards to a negligible temperature increase, as shown in the photothermal analysis. In the in vitro drug release test, a controlled DOX release by NIR light exposure was demonstrated. In addition, the DOX release kinetics could be enhanced by increasing the acidity of the environment.

## 4. Materials and Methods 

### 4.1. Materials

Propargyl alcohol (99%), 2–bromoisobutyryl bromide (97%), dimethylamino pyridine (DMAP, 99%), and copper wire (0.5 mm) were supplied by Alfa Aesar (Seoul, Korea). Glycidyl methacrylate, 1,4 dithio-D, L-threitol (DTT), Poly (ethylene glycol) methyl ether (average Mn 5000), trimethylamine, (97%), N, N′ dicyclohexylcarbodiimide (DCC), N,N,N′,N′,N′” pentamethyldiethylenetriamine (PMDETA), selenium powder, and sodium borohydride (NaBH_4_) were supplied by Sigma-Aldrich (Seoul, Korea). The 3,3’-diselanediyldipropanoic acid (DSeDPA) was prepared following the method of previous work [25]. Acetonitrile, dimethylformamide, dichloromethane, ethyl acetate, and tetrahydrofuran, hydrogen peroxide 37% were ordered from Duksan Pure Chemical (Seoul, Korea).

### 4.2. Preparation of the Diselenide Cross-Linkers

Propargyl alcohol (1.11 g, 20 mmol), DMAP (0.24 mg, 2 mmol), and DCC (4.13 g, 20 mmol) were mixed in 10 mL anhydrous tetrahydrofuran (THF) and cooled in an ice bath within 30 min. The cross-linker precursor i.e., DSeDPA (3.05 g, 10 mmol), in 5 mL THF was added slowly dropwise into the propargyl alcohol mixture and the solution was stirred in an ice bath within 30 min. The mixture solution was subsequently placed in a water bath at room temperature and continuously stirred for 12 h. The white precipitation of the dicyclohexylurea side-product was filtered out, and the solution containing the product was evaporated and dissolved in dichloromethane. The product was then washed with deionized water, dried using MgSO_4_, and finally concentrated using a rotary evaporator. A flash column chromatography SiO_2_ (EtOAc: Hex) was employed to purify the product, affording 2.60 g (68.5 % yield). ^1^H-NMR (600 MHz, CDCl_3_) δ: 2.77(t 4H, CH_2_CH_2_Se), 3.04(t, 4H, OCH_2_CH_2_), 3.80 (t, 4H, NCH_2_), 4.25 (t, 4H, OCH_2_), 6.73 (s, 4H, CH=CH). ^13^C-NMR (600 MHz, CDCl_3_) 171.51, 170.33, 133.88, 61.12, 36.87, 35.96, 22.07.

### 4.3. Preparation of PEO-Br Macroinitiator and PEO-b-PGMA

In a typical preparation of PEO-Br, 0.366g (3 mmol) of DMAP in 20 mL of dry methylene dichloride was mixed with 0.202 g (2 mmol) of TEA. The solution was transferred into a 250 mL three-neck round-bottom flask equipped with a condenser, dropping funnel, gas inlet/outlet, and a magnetic stirrer. After cooling to 0 °C, 1.115 g (5 mmol) of 2-bromoisobutyryl bromide in 20 mL of CH_2_Cl_2_ was added. To the yellow dispersion was added 10 g (2 mmol) of PEO in 100 mL of dry CH_2_Cl_2_ dropwise during 1 h under nitrogen; subsequently the temperature was allowed to rise to room temperature. The reaction was continued under stirring for 18 h. The solution was filtered, half of the solvent was evaporated, and the PEO–Br macroinitiator was precipitated in cold diethyl ether. After dissolution in absolute ethanol, the solution was stored overnight to recrystallize the product. The macroinitiator was filtered, washed with cold diethyl ether, and dried in vacuum. Yield 8.5 g (85%). ^1^H NMR (600 MHz, Chloroform-d) δ 4.29 (m, 2H), 3.67–351 (m, 452H), 3.34 (s, 3H), 2.14 (s, 2H), 1.90 (s, 6H).

In a typical preparation of PEO-*b*-PGMA, PEO-Br (0.52 g, 0.1 mmol), anisole (0.75 mL), and CuBr (0.014 g, 0.1 mmol) were mixed in a round-bottom flask then vigorously stirred under an atmosphere of N_2_. Subsequently, PMDETA (0.0174 g, 0.1 mmol) and GMA (0.25 g, 1.76 mmol) were added to the flask and continuously stirred at room temperature for 6 h. The product was dissolved into CH_2_Cl_2_ and purified by column chromatography. Finally, the product solution was precipitated in an excess amount of cold ethyl ether and dried under vacuum. The final product was collected and dried under vacuum at room temperature (2.68 g, yield 70%). ^1^H NMR (600 MHz, DMSO-d6) δ 4.37–4.24 (m, 28H), 3.82–3.69 (m, 28H), 3.56–3.46 3 (m, 456H), 3,24 (s, 3H), 3.23–3.18 (m, 28), 2.84–2.69 (dd, 56H), 2.05–1.60 (m, 56H), 1.03–0.73 (m, 84H). ^1^H NMR (600 MHz, DMSO-d_6_) δ 4.37–4.24 (m, 1H), 3.82–3.69 (m, 1H), 3.56–3.46 3 (m,16.2), 3,24 (s, 0.3H), 3.23–3.18 (m, 1H), 2.84–2.69 (dd, 2H), 2.05–1.60 (m, 2H), 1.03–0.73 (m, 3H)

### 4.4. Preparation of PEO-b-PGMA-N_3_

In a typical preparation, NaN_3_ (19.5 mg, 0.3 mmol), PEO-*b*-PGMA (0.10 g, 0.016 mmol of epoxide moieties), DMF (1 mL), and NH_4_Cl (16 mg, 0.3 mmol) were mixed into a round-bottom flask and then stirred at 50 °C for 24 h. The filtrates were subjected to a dialysis treatment (1 kDa, MW cutoff) against deionized water after removal of the insoluble salts for 24 h. The freshwater was periodically renewed in 4 h interval. The product was then dried in a freeze-dryer (yield 0.76 g, 76%). ^1^H NMR (600 MHz, DMSO-d_6_) δ 5.60–5.420 (m, 28H), 3.82–3.70 (board, 112H), 3.56–3.46 (m, 456H), 3,24 (s, 3H), 2.05–1.60 (m, 56H), 1.03–0.73 (m, 84H). ^1^H NMR (600 MHz, DMSO-d_6_) δ 4.37–4.24 (m, 1H), 3.82–3.70 (broad, 4H), 3.56–3.46 (m,15.2), 3,24 (s, 0.3H), 2.05–1.60 (m, 2H), 1.03–0.73 (m, 3H).

### 4.5. Preparation of Diselenide CCL Micelles 

In a typical preparation, PEO-*b*-PGMA-N_3_ (5.0 mg, 19.5 × 10^−3^ mmol of azide groups), DMF (3 mL), and the diselenide cross-linker (3.5 mg, 13 × 10^−3^ mmol) were mixed in a round-bottom flask. Subsequently, deionized water (30 mL) was poured into the solution, and vigorously stirred overnight. Sodium ascorbate (4 mg, 20 μmol) in 200 μL water and CuSO_4_.5H_2_O (4.5 mg, 18 μmol) in 200 μL water were added one by one and stirred at room temperature for 24 h. The solution was subsequently transferred into a bag for dialysis (1 kDa, MW cutoff) and the dialysis treatment was carried out against deionized water. Freshwater was replaced periodically in 4 h interval for 2 days. The micelle solution was passed through a 0.45 μm filter and dried in a freeze-dryer. ^1^H NMR (600 MHz, DMSO-d_6_) δ 5.65–4.97 (broad, 0.21H), 3.56–3.46 (m, 1H), 3.24–3.17 (s, 0.02H) 1.81–1.48 (m, 0.07H), 1.03–0.73 (m, 0.17H). The micelles loaded with DOX and ICG were prepared in the same manner with the addition of DOX (1 mg mL^−1^) at the initial step. The DOX loading efficiency was 61.6% and the DOX loading content was 9%. The ICG loading efficiency was 84.7% and the ICG loading content was 1.7%.

### 4.6. Determination of the Critical Micelles Concentration

The critical micelle concentration (CMC) was determined using a fluorescence spectrophotometer with pyrene as the probe, following a previously reported procedure [29]. A pyrene solution (6 × 10^−6^ M) in acetone was poured into tubes. Subsequently, the acetone was evaporated till completely dry at 60 °C. The polymer solutions with concentrations of 10–150 mg L^−1^ were poured into each tube so that the final pyrene concentration was 6 × 10^−7^ M. The solutions were then gently stirred at room temperature for 12 h. The fluorescence spectra were obtained using a fluorescence spectrophotometer (Hitachi F-4500, Hitachi High-Technologies Corporation, Tokyo, Japan) with an excitation wavelength of 333 nm and the emission fluorescence of 373 and 384 nm. The ratio of the peaks intensity at 373 nm (*I_373_*) to the peak at 384 nm (*I_384_*) was analyzed in graph to determine the CMC.

### 4.7. Drug Release Behavior

A dialysis method was used to study the in vitro drug release behavior of the DOX from the micelles of PEO-*b*-PFMA/ICG/DOX. Into a dialysis bag (13 kDa, MW cutoff) 3 mL of the micelles solution that was loaded with DOX was poured and subjected to a dialysis treatment against acetate buffer solution (0.01 M, pH 5.0) or PBS (0.01 M, pH 7.4) of mL. The dialysis process was carried out either with or without NIR light exposure (808 nm, 200 mW cm^−2^). The dialysis process was carried out in a water bath under continuous shaking at a rate of 85 rpm and a temperature of 37 °C. On the determined time intervals, 3 mL of the dialysate was taken for monitoring the DOX release and subsequently refilled with the same volume of the fresh acetate or PBS buffer solution. The concentration of the DOX that was released from the micelles was monitored using a UV-Vis spectrometer at a wavelength of 485 nm. The DOX release profile was determined using a cumulative method. Each experiment was conducted three times to ensure reproducibility.

### 4.8. Characterization

Thin-layer chromatography (TLC) analysis was performed using a Merck TLC silica gel 60 F254, aluminum-back TLC plates with an F254 fluorescent indicator, and 175–225 μm silica gel layer thickness. Column chromatography was done using silica gel with 60 Å porosity of 230–400 mesh. 

^1^H NMR spectra were measured on a JNM-ECP 600 (JEOL, JEOL Ltd., Tokyo, Japan) instrument. Chemical shifts were given in ppm using the following order: chemical shift, multiplicity, and coupling constants. The abbreviations of the multiplicity are as follows; s = singlet, d = doublet, t = triplet, m = multiplet. GPC was performed using an HP 1100 (Agilent Technologies INC., Santa Clara, CA, USA) apparatus equipped with a three PL gel column set (5 µm 104–103–102 Å) and a RI detector. GPC was calibrated using 12 polystyrene standards and *N,N*′-dimethylacetamide containing 50 mM LiCl was used as an eluent with a flow rate of 1 mL/min at 50 °C. 

Infrared spectra were recorded on an Agilent Cary 640 Fourier transform infrared (FT-IR, Agilent Technologies, Santa Clara, USA) spectrometer in a wavenumber region of 4000–400 cm^−1^ using a KBr pellet method. An Optizen POP spectrometer was employed to measure the UV-Vis spectra of each sample.

For transmission electron microscopy (TEM) measurement, a JEOL JEM-2010 (Hitachi Ltd., Tokyo, Japan) was employed. Prior to the measurement, samples were dispersed by sonication into acetonitrile and then deposited carefully on copper grids. The micelles particle size was measured using a dynamic laser light scattering (DLS, Zetasizer Nano ZS, Malvern Panalytical Ltd, Great Malvern, UK) at a wavelength of 633 nm. The intensity of scattered light was detected at 90° to an incident beam.
